# Contemporary educational and behavior change strategies improve dietary practices around a match in professional soccer players

**DOI:** 10.1080/15502783.2024.2391369

**Published:** 2024-08-12

**Authors:** Jennie L. Carter, David J. Lee, Jonathan S.J Fenner, Mayur K. Ranchordas, Matthew Cole

**Affiliations:** a Research Centre for Life and Sport Sciences (CLaSS), School of Health Sciences, Department of Sport and Exercise, Birmingham City University, Birmingham, UK; b Wolverhampton Wanderers Football Club, Wolverhampton, UK; c Advanced Wellbeing Research Centre, Health Research Institute, Sheffield Hallam University, Sheffield, UK; d Department of Sport & Exercise, Hartpury University, Gloucester, UK

**Keywords:** Soccer, nutrition, periodisation, intervention

## Abstract

**Background:**

The importance of nutrition in optimizing the health and performance of professional soccer players has been well established. Despite published practical recommendations for the dietary requirements for professional soccer players, many players fail to meet these guidelines. Thus, the primary purpose of this study was to assess the impact of targeted nutritional education and behavior change interventions on dietary intake in professional football players. Additionally, previous research within this population has reported elevations in resting metabolic rate (RMR) following match-play. Therefore, a further aim of this study was to examine whether any changes in dietary intake would influence RMR following match-play.

**Methods:**

Twenty players from the professional development phase in an English Premier League club (age: 18.4 ± 1.0 years; body mass: 76.1 ± 6.0 kg; stature: 1.80 ± 0.07 m) were randomly assigned to an “Intervention” (INT) group (*n* = 10), who received numerous nutritional education and behavior change interventions, or a “Control” (CON) group (*n* = 10), who received no nutrition support. Dietary intake was assessed daily throughout the match-week (Match Day (MD)-2, MD-1, MD, MD + 1, and MD + 2), whilst RMR was assessed on MD-1, MD + 1, and MD + 2. Statistical analyses on the intervention effects on dietary intake and RMR were carried out using a two factor (group and day) analysis of variance (ANOVA) with a subsequent Bonferroni post-hoc test.

**Results:**

Mean energy (3393 ± 852 vs. 2572 ± 577 kcal · day^−1^) and CHO (5.36 ± 1.9 vs. 3.47 ± 1.1 g · kg^−1^ BW · day^−1^) intake was significantly higher (*p* < 0.001) in the INT vs. CON group. Furthermore, the INT group implemented nutrition periodization practices as CHO intake was significantly increased on MD-1 (7.0 ± 1.7 g · kg^−1^ BM · day^−1^), MD (7.1 ± 1.4 g · kg^−1^ BM · day^−1^) and MD + 1 (5.1 ± 0.8 g · kg^−1^ BM · day^−1^). However, the CON group did not periodize their CHO intake and failed to meet the CHO recommendations on MD-1, MD, and MD + 1 (<4 g · kg^−1^ BM · day^−1^). Compared to MD-1, the RMR increased on MD + 1 and MD + 2 in both groups, although it was only statistically significant for the INT group (MD + 1 =  +243 kcal · day^−1^; MD + 2 =  +179 kcal · day^−1^).

**Conclusions:**

The implementation of targeted nutritional education and behavior change interventions resulted in improved dietary practices in professional football players and enabled better adherence to recommended guidelines. However, despite this, RMR was still elevated in the 24–48 h following match play. Thus, in order to optimize recovery, this finding further reinforces the need for professional football players to adopt strategies to meet energy, and particularly CHO, requirements in the acute period following a match in order to account for this increase in energy requirement.

## 1. Introduction

The importance of nutrition in optimizing the health and performance of professional football players has been well established [1]. Sufficient energy intake (EI) is critical to support the demands of training and competition, growth and development [2], promote training adaptations, and optimize performance [3]. Inadequate nutritional intake can lead to impaired physiological function, increased risk of fatigue, illness and injury, and maladaptation to the training stimulus [4].

Despite the published practical recommendations for dietary requirements for professional football players [1], many players fail to meet these guidelines [5,6]. Although professional football players are typically able to meet daily protein and fat intake recommendations [7], energy intakes have been reported to be inadequate to meet the demands of training and competition [8,9]. Additionally, daily carbohydrate (CHO) intakes are inadequate to optimize fueling and recovery [7], specifically, the day before, and the day following a match [5,6,9]. This is of concern given the role of CHO for optimal performance and recovery [1]. Additionally, as resting metabolic rate (RMR) has previously been reported to be significantly elevated in the day following a competitive match [6], not meeting nutrition recommendations following a match could be detrimental to recovery. Collectively, these findings highlight the need for the development of effective strategies that can better enable professional football players to meet their nutritional requirements.

In order to positively influence the dietary behavior of professional football players, a performance nutritionist is required to implement interventions. However, to ensure these are effective, dietary behaviors need to be fully understood first [10]. Understanding the key influencers of an athlete’s behavior enables us to identify which aspects require change to allow positive dietary behavior to arise. Various models for understanding behavior exist and are utilized to develop an understanding of the determinants of dietary behaviors. Michie et al. [11] developed a behavior change model, known as the Capability, Opportunity, Motivation – Behavior (COM-B) model. COM-B is a meta-theory that deviates our focus from individual blame to shared responsibility for behavior change, in comparison to preexisting behavior change theories which tend to focus solely on the individual (ie the Social Cognitive Theory and Health Belief Model). The COM-B model is deemed the most appropriate for assessing key influencers of an athlete’s behavior as it is suggested that other models inadequately explain variations in complex human behavior [12]. Unlike these other theories, the COM-B incorporates the impact of the social and physical environment, which have been highlighted as important influencers of athlete behavior due to the complex environments within which athletes typically operate [10,13]. It is suggested that capability, opportunity, and motivation interact to generate behavior change. “Capability” is defined as “the individuals” psychological and physical capacity to engage in an activity; “Opportunity” incorporates all the factors, besides the individual, which make the behavior possible or prompt it (eg social or physical factors); and “Motivation” is defined as “all the brain processes that direct behaviour” [11]. If more detail is required to interpret the behavior, the Theoretical Domains Framework (TDF) can be used to elaborate on the COM-B components which is composed of 14 domains combined from 128 theoretical constructs taken from 33 theories of behavior change [14]. Currently, limited research has investigated dietary behaviors in athletes, demonstrating many factors may influence a player’s food choices, such as cultural, religious, ethical, and food preferences [15–18]. To date, only one study has investigated the barriers and enablers to nutritional adherence within professional football players [19], which utilized the COM-B model. More specifically, Carter et al. [19] reported that nutritional knowledge, cooking skills, training venue food provision, nutritionist accessibility and approachability, living status, performance implications, and role modeling were seven key themes identified relating to the players’ barriers and enablers to nutritional adherence. These findings are similar to those reported in other sporting populations [15,16,20–24] which suggest that these common issues are shared across different sports.

**Figure 1.**
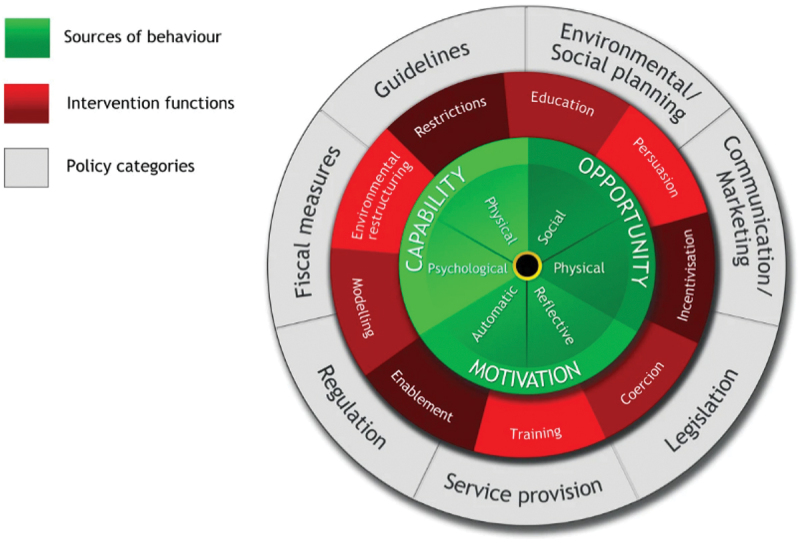
The behavior change wheel Michie et al., (2011).

Following an understanding of the factors influencing dietary behaviors, these outcomes can be subsequently linked to the Behavior Change Wheel (BCW) [11] to help design evidence-based behavior change strategies, which has previously been utilized within the field of sports nutrition to design successful nutritional interventions [13]. The COM-B model forms the center of the BCW, around which are positioned nine “intervention functions” designed to address deficits in one or more of the COM-B constructs. Furthermore, seven “policy categories” are placed around the outside of the BCW which are designed to support the implementation of the interventions (Figure 1). To the authors' knowledge, there has been no previous research which has directly assessed the impact of evidence-based strategies to change the dietary behavior of professional football players. Therefore, utilizing the findings of Carter et al. [19], the primary aim of this study was to design a series of behavior change strategies to determine whether these would positively influence the dietary practices of professional football players in an applied environment. A secondary aim of this study was to assess whether adherence to recommended dietary guidelines could ameliorate some of the previously reported elevations in resting metabolic rate (RMR) in the days following a competitive match [6]. The hypothesis of the study was that the implementation of the aforementioned behavior change strategies, underpinned by previous research, would positively influence dietary intake in professional soccer players. Additionally, we hypothesize that based upon previous findings, RMR will continue to increase following a competitive match, although a novel aspect of this study is to establish if this is influenced by player adherence to nutritional recommendations.

## 2. Methods

### 2.1. Participants

A convenient sample of 20 professional football players (age: 18.4 ± 1.0 years; body mass: 76.1 ± 6.0 kg; stature: 1.80 ± 0.07 m; defenders *n* = 10; midfielders *n* = 5; forwards *n* = 5) from the Professional Development Phase of a Category 1 English Premier League club were recruited for this study. This included 18 U23s players and 2 U18s players. Inclusion criteria included fully fit players (injured players were excluded) who were playing full match minutes (90 min played by all players). All participants gave their written informed consent to participate in the investigation following approval from the Faculty Academic Ethics Committee at Birmingham City University, UK (Approval Number: Carter/#11370/sub1/R(B)/2022).

### 2.2. Anthropometric measures

Body mass (kg; SECA, model-875, Hamburg, Germany) and stature (m; SECA, model-217, Hamburg, Germany) were measured on the first day of assessment, according to The International Society for the Advancement of Kinanthropometry (ISAK) guidelines [25], in the morning with minimal clothing and items such as jewelry removed.

### 2.3. Research design

Data was collected in-season during a typical training and match week between February and May 2023 (see Table 1 for a typical training week schedule).

**Table 1.** An overview of the pitch-based and match schedule for each squad.

	Monday	Tuesday	Wednesday	Thursday	Friday	Saturday	Sunday
U23	Match	Recovery	Day off	Training	Training	Training	Training
(am)		11:00 – 12:00		10:45 – 12:30	10:45 – 12:30	10:45 – 12:30	10:45 – 12:30
(pm)	19:00 Kick Off			Gym 14:00-15:00		Gym 14:00-15:00	
U18	Training	Training	Day off	Training	Training	Match	Day off
(am)	10:45 – 12:30	10:45 – 12:30		10:45 – 12:30	10:45 – 12:30	11:00 Kick Off	
(pm)		Gym 14:00-15:00		Gym 14:00-15:00			

Timepoints throughout the study are described relatively to match day (MD) using ± symbols for days before (-) or after (+) MD. The RMR was measured on MD-1, MD + 1, and MD + 2 as we have previously reported increases in RMR in the days following a match when compared to the days prior to the match [6]. Dietary intake was measured on MD-2, MD-1, MD, MD + 1, and MD + 2. The 20 players were randomly assigned to an “Intervention” (INT) (*n* = 10; U23s = 9, U18s = 1) or “Control” (CON) (*n* = 10; U23s = 9, U18s = 1) group. The CON group did not receive any nutritional support from the Club’s nutritionist throughout this phase. The INT group received various educational, and behavior change strategies mapped to the BCW [11]. These included a range of intervention strategies such as: an educational workshop on fueling and recovery; a cooking workshop; prompts and infographics via WhatsApp; the nutritionist being present at mealtimes within the training ground; a motivational video from a senior player; and post-match recovery snack bag provision (see Table 2 for more detailed outline of these strategies and timeline of their implementation). These interventions were based on findings from the previous study, where we identified the key barriers and enablers of adherence to nutritional recommendations in professional football players [19] utilizing the COM-B model [11]. At the end of the intervention period, players in the INT group completed an online questionnaire evaluate the efficacy of the intervention in positively changing their dietary behaviors.

**Table 2.** A summary of the INT group educational and behavior change strategies mapped to the BCW Michie et al., (2011), (italics font in brackets demonstrate the intervention functions of the BCW to which the strategies implemented align).

Timepoint	Intervention
1 week before data collection	Individual cooking workshop (1 h) *(Training)* Educational workshop on nutritional periodization throughout the competitive week, focusing on match day fueling and recovery *(Education)*
MD-2	1^st^ Team player motivational video sent via WhatsApp (discussing nutritional habits around a match) *(Modelng)* Nutritionist present at mealtimes within training ground *(Persuasion)*
MD-1	Educational infographic sent via WhatsApp on nutritional preparation for the match *(Education)* WhatsApp prompts from Nutritionist at mealtimes to encourage high CHO intake *(Persuasion)* Nutritionist present at mealtimes within training ground *(Persuasion)*
MD	Educational infographic sent via WhatsApp on match day nutrition (fueling and recovery) *(Education)* WhatsApp prompts from Nutritionist at mealtimes to encourage high CHO intake *(Persuasion)* Nutritionist present at mealtimes within training ground *(Persuasion)*
MD + 1	Recovery snacks bag provided in the morning (included four high CHO snacks & drinks) *(Enablement)* WhatsApp prompts from Nutritionist at mealtimes to encourage high CHO intake *(Persuasion)* Nutritionist present at mealtimes within training ground *(Persuasion)*

Key: MD = match day.

### 2.4. Assessment of energy and macronutrient intake

EI was assessed using the remote food photographic method (RFPM), known as “Snap-N-Send” which has been shown to be a valid and reliable dietary assessment tool in athletes [26] and utilized in the previous research [5]. Additionally, players in this study were well accustomed to this method given it is common practice within the professional football environment in which they are based. Dietary intake was recorded for 5 days (MD-2, MD-1, MD, MD + 1, and MD + 2) to enable assessment of how EI may vary across the competitive week. On the day before data collection, players were informed by the lead researcher (a Sport and Exercise Nutrition Register (SENr) Practitioner) how to accurately and comprehensively complete the Snap-n-Send tool, ensuring accurate recording of the time of food consumption, amount (weighed amount or household measures such as tablespoons, teaspoons, cups), and description of food (cooking & preparation methods, ingredients, and brands). Photographs were sent through an instant messaging application (WhatsApp, Dublin, Ireland). If the photo or food descriptions were unclear, the player would be contacted in real time to clarify details which helped improve the accuracy of food diaries. Where food was consumed within the training ground, the lead researcher assisted participants with dietary recording (descriptions, investigating cooking methods with chefs, etc.). If there was any food or drink left following consumption, participants were instructed to send a photo of what had not been consumed so that dietary intakes could be adjusted. A 24-hr recall was also undertaken with each participant each morning to cross-reference, check for missing data, confirm amounts, and seek further clarity if required, which was then added to the participants’ record.

Energy and macronutrient intake was obtained using a professional dietary analysis software (Nutritics Ltd, v5, Ireland). All the dietary information was inputted into the software by the lead researcher and checked by an additional member of the research team to ensure consistency. The inter-rater reliability is determined via an independent t-test. No significant differences were observed between researchers for energy (*p* = 0.826, 95% CI − 500 to 602 kcal), CHO (*p* = 0.799, 95% CI − 17.4 to 22.2 g), or protein (*p* = 0.127, 95% CI −27.8 to 3.9 g) intake. Meals were either consumed at: the club’s training ground (where a buffet breakfast, lunch, pre- and post-match meals, drinks, snacks, and supplements were provided); a hotel (where players may be on match day); on the coach during travel on match day or; the players’ home environment or restaurants if they chose to eat out. For the meals provided at the training ground, at the hotel or on the coach, menus were provided on a buffet-style basis. All meals were consumed ad libitum by players during the study.

### 2.5. Resting metabolic rate

RMR was measured a total of three times for each participant. All measures were undertaken at the same time between 7.30 and 9.30 am and the players arrived at the training ground following an overnight fast, with their last meal at least 8 h prior to the measurement. Participants abstained from caffeine, alcohol and nicotine overnight, and avoided physical activity for 14 h prior to measurement (Fullmer et al., 2015). A private, quiet room was utilized to conduct the measurements with a temperature maintained at an ambient condition of 20–22°C (Fullmer et al., 2015). Players lay in a comfortable supine position and were reminded to stay awake throughout the assessment. Prior to the measurement, players rested for 20 min (Fullmer et al., 2015). Following this, the RMR was measured for 20 min. The ventilated hood was located over the participant’s head, and expired gas was collected via the dilution canopy method (Vyntus CPX canopy, CareFusion, Hoechberg, Germany). A visual check every 5 min ensured no gas was escaping. The gas analyzer was calibrated daily using the manufacturer’s automated flow and digital volume transducer calibration (15.92% O_2_ and 5.03% CO_2_). Following the best practice guidelines, the first 5 min of measurements were discarded [27]. Measurements were subsequently recorded for 15 min continuously at 10-s intervals, energy expenditure (kcal · day^−1^) was calculated using the Weir equation [28]. The coefficient of variance for our protocol was measured at 1.47% for RMR, which was similar to our previous work using identical methods within this population (1.59%; [6]).

### 2.6. Statistical analysis

The statistical analysis was performed using SPSS Software Version 28.0 (SPSS Inc., Chicago, IL, USA). Statistical analyses on the intervention effects on dietary intake and RMR were carried out using a two factor (group and day) analysis of variance (ANOVA) with a subsequent Bonferroni post-hoc test. For all the analyses, a statistical significance was set at *p* < 0.05. All data are reported as Mean ± SD unless otherwise stated.

## 3. Results

### 3.1. Energy intake

**Figure 2.**
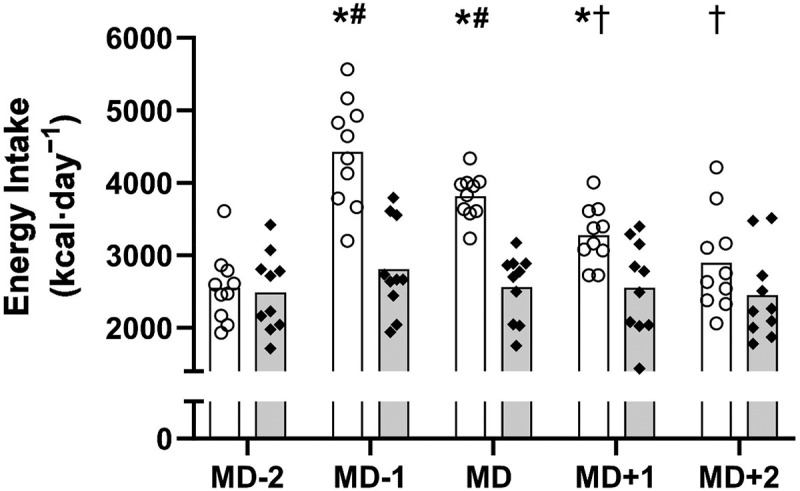
Daily energy intake (kcal · day^−1^) across the competitive match week (mean). White bars = intervention group. Grey bars = control group. O = individual players in the intervention group. ♦ = individual players in the control group. *significantly higher than control MD-1 and MD (*p* < 0.001) and MD + 1 (*p* = 0.008). ^#^Significantly higher than intervention MD-2 (*p* < 0.001). ^†^Significantly lower than intervention MD-1 (MD + 1: *p* = 0.008; MD + 2: *p* < 0.001). Key: MD = match day.

Daily absolute EI is displayed in Figure 2.

The overall mean EI was significantly higher in the INT vs. CON group, irrespective of individual days (3393 ± 852 vs. 2572 ± 577 kcal · day^−1^, respectively, *p* < 0.001). The EI in the INT group was significantly higher than the CON group on MD-1 (4423 ± 738 vs. 2807 ± 642 kcal · day^−1^, respectively, *p* < 0.001) MD (3815 ± 310 vs. 2561 ± 464 kcal · day^−1^, respectively, *p* < 0.001); and MD + 1 (3277 ± 408 vs. 2553 ± 646 kcal · day^−1^, respectively, *p* = 0.008). There were no significant differences in the EI across any of the days within the CON group (*p* = 1.000), however the INT group significantly increased the EI on MD-1 (+1870 kcal · day^−1^: *p* < 0.001) and MD (+1263 kcal · day^−1^: *p* < 0.001) in comparison to MD-2. Furthermore, the EI on MD + 1 (−1147 kcal · day^−1^: *p* = 0.008) and MD + 2 (−1528 kcal · day^−1^: *p* < 0.001) significantly reduced compared to MD-1 in the INT group.

### 3.2. CHO intake

**Figure 3.**
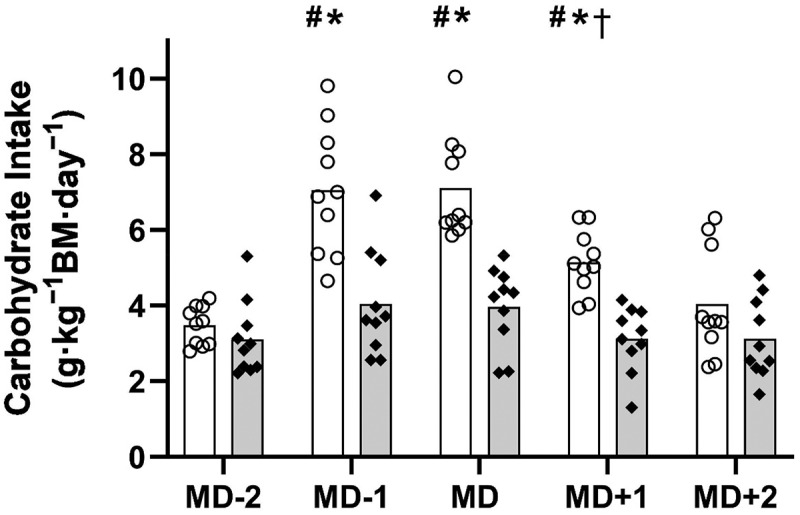
Daily relative carbohydrate intake (g · kg^−1^ BM · day^−1^) across the competitive match week (mean). White bars = intervention group. Grey bars = control group. O = individual players in the intervention group. ♦ = individual players in the control group. *significantly higher than control MD-1, MD and MD + 1 (*p* < 0.001). ^#^Significantly higher than intervention MD-2 (*p* < 0.001). ^†^Significantly lower than MD (*p* < 0.001). Key: MD = match day.

Daily relative CHO intake is displayed in Figure 3.

The overall mean CHO intake was significantly higher in the INT vs. CON group, irrespective of individual days (5.36 ± 1.9 vs. 3.47 ± 1.1 g · kg^−1^ BM · day^−1^, respectively, *p* < 0.001). The CHO intake in the INT group was significantly higher than the CON group on MD-1 (7.0 ± 1.7 vs. 4.0 ± 1.4 g · kg^−1^ BM · day^−1^, respectively, *p* < 0.001); MD (7.1 ± 1.4 vs. 4.0 ± 1.1 g · kg^−1^ BM · day^−1^, respectively, *p* < 0.001); and MD + 1 (5.1 ± 0.8 vs. 3.1 ± 0.9 g · kg^−1^ BM · day^−1^, respectively, *p* < 0.001). There were no significant differences in CHO intake across any of the days within the CON group (*p* > 0.05), however the INT group significantly increased CHO intake on MD-1 (+3.6 g · kg^−1^ BM · day^−1^: *p* < 0.001) MD (+3.6 g · kg^−1^ BM · day^−1^: *p* < 0.001) and MD + 1 (+1.7 g · kg^−1^ BM · day^−1^: *p* < 0.001) in comparison to MD-2. There were no significant differences between MD-2 and MD + 2 CHO intake in the INT group (*p* = 1.000), although MD + 1 CHO intake was significantly lower than MD (- 2.0 g · kg^−1^ BM · day^−1^: *p* < 0.001).

### 3.3. Protein intake

**Figure 4.**
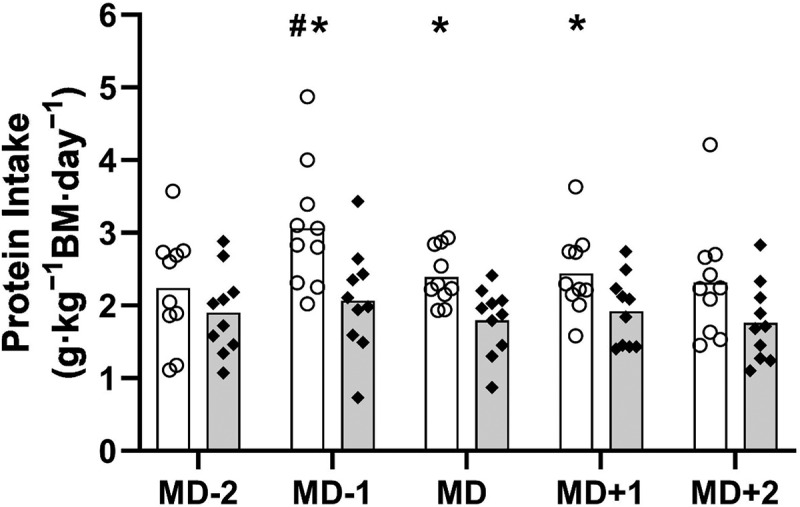
Relative protein intake (g · kg^−1^ BM · day^−1^) across the competitive match week (mean). Key: MD = match day. White bars = intervention group. Grey bars = control group. O = individual players in the intervention group. ♦ = individual players in the control group. *significantly higher than control MD-1 (MD-1: *p* = 0.012; MD: *p* = 0.005; MD + 1: *p* = 0.042). ^#^Significantly higher than intervention MD-2 (*p* = 0.036). Key: MD = match day.

Daily relative protein intake is displayed in Figure 4.

The overall mean protein intake was significantly higher in the INT vs. CON group, irrespective of individual days (2.5 ± 0.7 vs. 1.9 ± 0.6 g · kg^−1^ BM · day^−1^, respectively, *p* = 0.006). Protein intake in the INT group was significantly higher than the CON group on MD-1 (3.1 ± 0.9 vs. 2.1 ± 0.7 g · kg^−1^ BM · day^−1^, respectively, *p* = 0.012); MD (2.4 ± 0.4 vs. 1.8 ± 0.5 g · kg^−1^ BM · day^−1^, respectively, *p* = 0.005); and MD + 1 (2.4 ± 0.6 vs. 1.9 ± 0.5 g · kg^−1^ BM · day^−1^, respectively, *p* = 0.042). There were no significant differences in protein intake across any of the days within the CON group (*p* = 1.000), however the INT group had a significantly higher protein intake on MD-1 vs. MD-2 (3.1 ± 0.9 g · kg^−1^ BM · day^−1^ vs. 2.2 ± 0.8 g · kg^−1^ BM · day^−1^, respectively, *p* = 0.036).

### 3.4. Resting metabolic rate

**Figure 5.**
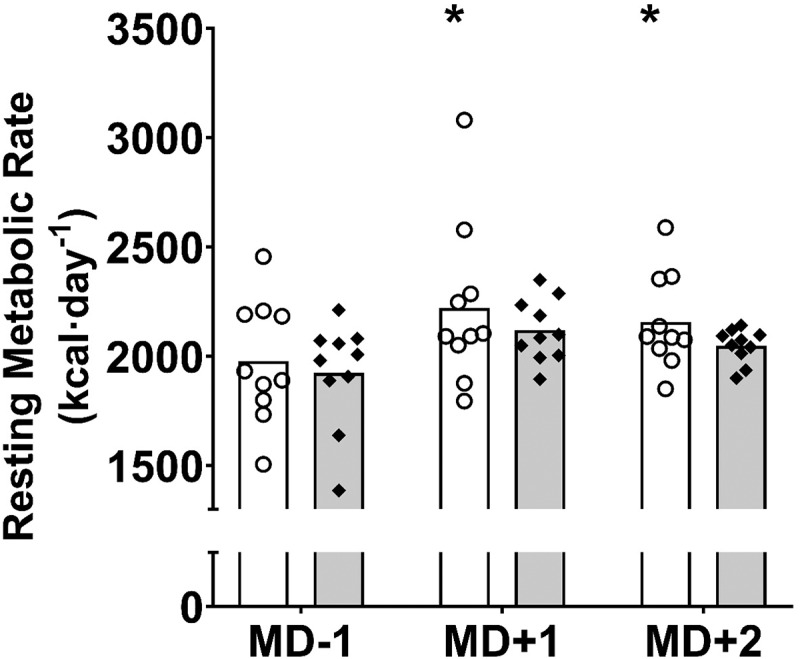
RMR (kcal · day^−1^) across the competitive match week (mean). White bars = intervention group. Grey bars = control group. O = individual players in the intervention group. ♦ = individual players in the control group. *significantly higher than intervention MD-1 (MD + 1: *p* = 0.015; MD + 2: *p* = 0.018). Key: MD = match day.

Daily absolute RMR is displayed in Figure 5.

RMR following the match was significantly higher than the pre-match values in the INT group (MD + 1 = 2220 ± 372 kcal · day^−1^ vs. MD-1 = 1976 ± 280 kcal · day^−1^, +243 kcal · day^−1^, *p* = 0.015; MD + 2 = 2156 ± 217 kcal · day^−1^ vs. MD-1, +179 kcal · day^−1^, *p* = 0.018). Similarly, in the CON group, the RMR increased following the match, but did not achieve statistical significance (MD + 1 = 2118 ± 143 kcal · day^−1^ vs. MD-1 = 1923 ± 242 kcal · day^−1^, +195 kcal · day^−1^, *p* = 0.057; MD + 2 = 2047 ± 78 kcal · day^−1^ vs. MD-1, +124 kcal · day^−1^, *p* = 0.134). In comparison to MD + 1, RMR did not significantly decrease by MD + 2 in the INT (MD + 1 vs. MD + 2, −64 kcal · day^−1^, *p* = 1.000) or CON group (MD + 1 vs. MD + 2, −71 kcal · day^−1^, *p* = 0.903). There were no significant differences between the groups on any of the days (MD-1, MD + 1, and MD + 2: *p* > 0.005).

### 3.5. Efficacy of intervention strategies

At the end of the intervention period, 60% of the players (*n* = 6) stated that their dietary behaviors had improved as a result of the intervention strategies, whilst 40% of the players (*n* = 4) felt that this had remained unchanged. Player ratings of the effectiveness of the different educational and behavior change strategies are shown in Table 3.

**Table 3.** INT group player ratings of the effectiveness of educational and behavior change strategies in improving their dietary intake.

Intervention	Strongly disagree	Somewhat disagree	Neither agree nor disagree	Somewhat agree	Strongly agree
Educational workshop on fuelling and recovery	-	-	2	5	3
Recording dietary intake via “Snap-N-Send”	-	-	3	6	1
WhatsApp prompts from the sports nutritionist	-	-	-	3	7
Nutritionist being present at mealtimes within the training ground	-	-	3	6	1
Motivational video from first team player	-	-	7	3	-
Cooking workshop	-	-	7	3	-

The WhatsApp prompts from sports nutritionists were the highest rated intervention, with 70% of players indicating that they strongly agreed that this had a positive impact on their dietary behaviors. Conversely, the motivational video from a first team player and the practical cooking workshop were reported to be least effective, with 70% of players reporting in each case that these had little impact on their dietary intake.

## 4. Discussion

The purpose of the current study was to design a series of behavior change strategies to determine whether these would positively influence the dietary practices of professional football players, in addition to assessing whether adherence to dietary guidelines could ameliorate some of the previously reported elevations in RMR in the days following a competitive match. Interestingly, on average, players within the INT group consumed significantly more energy (+821 kcal · day^−1^) and carbohydrate (+1.9 g · kg^−1^ BM · day^−1^) than players in the CON group. Additionally, players in the CON group did not periodize their energy or CHO intake across any of the days, whilst players in the INT group demonstrated evidence of nutritional periodization throughout the week. In relation to MD-1 and MD specifically, the INT group consumed significantly more CHO (~7.0 g · kg^−1^ BM · day^−1^) in comparison to a training day (~3.5 g · kg^−1^ BM · day^−1^), which is in line with pre-match recommendations of 6–8 g · kg^−1^ BM · day^−1^ [1]. However, the CON group failed to meet these recommendations (~4.0 g · kg^−1^ BM · day^−1^), which is in support of previous research highlighting inadequate CHO intakes on MD-1 (~3.9 [6] and <5 g · kg^−1^ BM · day^−1^ [5], respectively) and MD (~3.6 [6] and 5.1 g · kg^−1^ BM · day^−1^ [5], respectively). The findings from our study suggest the interventions were effective in supporting players to increase CHO intake on these critical days to optimize fueling for the match. Additionally, our findings suggest that when players do not receive targeted behavior change strategies, they consume considerably lower CHO intakes compared to recommendations, which highlights the importance of targeted nutrition support. Although players in the INT group significantly increased their CHO intake on MD + 1 (~5.2 g · kg^−1^ BM · day^−1^) compared to a training day, in addition to being significantly higher than the CON group (~3.1 g · kg^−1^ BM · day^−1^), these intakes were below the recommended CHO intake of 6–8 g · kg^−1^ BM · day^−1^ for MD + 1 [1]. Therefore, although players in the INT group appeared to implement adequate fueling prior to the match, there is evidently a lack of implementation of maintaining high CHO intakes in recovery from match play which has also been reported in the previous research in professional football [5,6]. On MD + 1, Anderson et al. [5] reported CHO intakes of <4 g · kg^−1^ BM · day^−1^ and Carter et al. [6] reported CHO intakes of ~3.6 g · kg^−1^ BM · day^−1^. These sub-optimal CHO intakes may have negative implications for subsequent training sessions or matches, especially during periods of fixture congestion as CHO intakes are even more crucial during this period to support recovery [29].

The mean daily protein intake was significantly higher in the INT (2.5 g · kg^−1^ BM · day^−1^) compared to the CON (1.9 g · kg^−1^ BM · day^−1^) group, however both groups were within the daily protein intake requirements of 1.6–2.2 g · kg^−1^ BM · day^−1^ [1]. These reported daily protein intakes are similar to that of previous research within professional football players who reported protein intakes of ~1.7 g · kg^−1^ BM · day^−1^ [9] and ~2.5 g · kg^−1^ BM · day^−1^ [5]. Therefore, our findings suggest that players are capable of meeting protein intake guidelines regardless of whether they receive nutritional support. This may suggest that nutrition practitioners working in professional football should focus their support on areas of greater concern, namely ensuring that players consume sufficient CHO and total energy.

As previously mentioned, limited research exists on the impact of nutritional interventions on dietary intake in athletes. Typically, existing research focuses on one specific intervention, for example Heikkilä et al. [30] assessed the impact of nutrition education on dietary intake in endurance athletes and found that nutrition education intervention alone was not enough to change dietary intake, specifically in relation to energy and CHO intake. Likewise, Garthe et al. [31] also found no improvement in dietary intakes or body composition in elite athletes following nutrition counseling alone. Collectively, these studies may suggest that a variety of nutritional interventions targeting various behaviors are required to result in dietary intake improvements, as typically, behaviors do not occur in isolation. It is important to recognize that athletes are individuals and will behave differently in response to intra-personal, inter-personal, and external factors [32]. The fact that our study incorporated a range of intervention strategies may explain why we observed positive changes in dietary intake across the group as there were clear differences in individual player perceptions of the most impactful intervention strategy (Table 3). Our findings are supported by the work of Costello et al. [13] who also utilized the COM-B and BCW in the design of various nutritional interventions in a case study of a professional rugby player and reported positive outcomes in dietary intake. Whilst that study did not seek to assess the efficacy of the different nutritional interventions, our data would suggest that prompts from the sport nutritionist (via a mobile instant messaging app - WhatsApp) is the most effective intervention strategy to improve the dietary behaviors in professional football players. However, given that our study is the first of its kind to evaluate the efficacy of such interventions within this population, we advise caution before recommending that this is adopted as a widespread practice and suggest that future research should seek to validate these findings further.

In terms of RMR, our findings support that of previous research, also undertaken in professional football players, which has also shown that RMR is elevated the day following competitive match play [6]. Additionally, these findings are consistent with that of Hudson et al. [33] who reported similar increases in RMR in the 24–48-h post-match period in professional rugby players. Hudson et al. [33] proposed that the elevated RMR may be a consequence of raised energy requirement due to a high physical load inducing the degradation and resynthesis of damaged muscle fibers [34]. Carter et al. [6] also suggested that the high physical load on MD and increased muscle soreness following the match may have influenced the reported elevations in RMR. In the present study, whilst elevations in the RMR were observed in both groups, these were only statistically significant for the INT group (MD + 1: *p* = 0.015; MD + 2: *p* = 0.018). As this study was undertaken in an applied professional football environment, where sample sizes are typically low as access to players is often limited, it is likely that the small sample size of 10 players may be a potential factor to explain why the control group did not quite achieve statistical significance (MD + 1: *p* = 0.057; MD + 2: *p* = 0.081). Interestingly, the mean increases in RMR for the CON group on MD + 1 (+195 kcal · day^−1^) and MD + 2 (+124 kcal · day^−1^) were similar to that of the INT group and thus further research may need to utilize larger sample sizes in order to achieve statistical significance. Nonetheless, from a practical perspective, our study is the first of its kind to show that even when players meet CHO intake recommendations in the day prior to and day of a match, RMR is still elevated in the 24–48 h following match play. Thus, in order to optimize recovery, this finding further reinforces the need for professional football players to adopt strategies to meet energy, and particularly CHO, requirements in the acute period following a match in order to account for this increase in energy requirement.

### 4.1. Strengths and limitations

Whilst the findings of this study are limited to one professional Premier League football club, it is likely that training and match schedules are similar in other clubs of a comparable standard and thus it would be interesting to establish whether our observations are replicated across the professional football environment. A key strength of this research was the fact it was undertaken by a real-world Premier League football club with professional players. Additionally, to date, no previous research has investigated the effect of behavior change strategies on dietary intake and assessed RMR simultaneously specifically within professional football. A limitation is the potential of participants under-reporting dietary intake on the RFPM and 24-h recall. However, as outlined in the methods, robust steps were undertaken to minimize this. Additionally, a further limitation is that the study sample included participants from two different age groups (U23s and U18s), who had minor differences in training and match schedules which may impact upon the findings. Thus, future research should seek to assess isolated age groups to enable comparisons of findings between these groups. Finally, whilst our study indicated that some strategies were likely more effective in inducing positive dietary behaviors, it is important to acknowledge that this data was obtained from a limited number of players. Future research should seek to recruit larger sample sizes to allow the comparison of different behavior change strategies and the impact this has on dietary behavior, in addition to enabling comparisons between playing positions. Furthermore, similar studies are required for female players to explore whether these issues are evident within that population given that they are significantly underrepresented in the literature. Collectively, this would help to support nutrition practitioners in designing effective nutrition programs for professional football players.

## 5. Conclusion

In conclusion, our data demonstrated that the implementation of nutrition strategies based on the COM-B and BCW models resulted in positive dietary behaviors, specifically in increasing EI, and meeting CHO requirements in the day prior to, and day of, a match. We report that when players do not receive targeted behavior change strategies, they consume considerably lower CHO intakes compared to recommendations. Additionally, our data shows that even when players meet CHO intake recommendations on the day prior to and the day of a match, RMR is still elevated. Therefore, nutrition practitioners should focus on implementing sound, evidence-based behavior change interventions to promote effective fueling and recovery nutrition practices in the day prior to the match, MD and within the 1–2 days following a match.

## References

[1] Collins J, Maughan RJ, Gleeson M, et al. UEFA expert group statement on nutrition in elite football. Current evidence to inform practical recommendations and guide future research. Br J Sports Med. 2021;55(8):416. doi: 10.1136/bjsports-2019-101961 33097528

[2] Spear BA. Adolescent growth and development. J Am Diet Assoc. 2002;102(3):S23–16. doi: 10.1016/s0002-8223(02)90418-9 11902385

[3] Petrie HJ, Stover EA, Horswill CA. Nutritional concerns for the child and adolescent competitor. Nutrition. 2004;20(7–8):620–631. doi: 10.1016/j.nut.2004.04.002 15212744

[4] Mountjoy M, Sundgot-Borgen J, Burke L, et al. The IOC consensus statement: beyond the female athlete triad–relative energy deficiency in sport (RED-S). Br J Sports Med. 2014;48(7):491–497. doi: 10.1136/bjsports-2014-093502 24620037

[5] Anderson L, Orme P, Naughton RJ, et al. Energy intake and expenditure of professional soccer players of the English premier league: evidence of carbohydrate periodization. Int J Sport Nutr Exerc Metab. 2017;27(3):228–238. doi: 10.1123/ijsnem.2016-0259 28050927

[6] Carter J, Lee DJ, Perrin CG, et al. Significant changes in resting metabolic rate over a competitive match week are accompanied by an absence of nutritional periodization in male professional soccer players. Int J Sport Nutr Exerc Metab. 2023;33(6):349–359. doi: 10.1123/ijsnem.2023-0069 37734739

[7] Steffl M, Kinkorova I, Kokstejn J, et al. Macronutrient intake in soccer players-a meta-analysis. Nutrients. 2019;11(6):1305. doi: 10.3390/nu11061305 31181835 PMC6627126

[8] Briggs MA, Cockburn E, Rumbold PLS, et al. Assessment of energy intake and energy expenditure of male adolescent academy-level soccer players during a competitive week. Nutrients. 2015;7(10):8392–8401. doi: 10.3390/nu7105400 26445059 PMC4632420

[9] Brinkmans NYJ, Ledema N, Plasqui G, et al. Energy expenditure and dietary intake in professional football players in the Dutch premier league: implications for nutritional counselling. J Sports Sci. 2019;37(24):2759–2767. doi: 10.1080/02640414.2019.1576256 30773995

[10] Bentley MRN, Mitchell N, Backhouse SH. Sports nutrition interventions: a systematic review of behavioural strategies used to promote dietary behaviour change in athletes. Appetite. 2020;150:104645. doi: 10.1016/j.appet.2020.104645 32112958

[11] Michie S, Stralen MM, West R. The behaviour change wheel: a new method for characterising and designing behaviour change interventions. Implement Sci. 2011;6(1). doi: 10.1186/1748-5908-6-42 PMC3096582 21513547

[12] Coulson NS, Ferguson MA, Henshaw H, et al. Applying theories of health behaviour and change to hearing health research: time for a new approach. Int J Audiol. 2016;55(sup3):S99–S104. doi: 10.3109/14992027.2016.1161851 27138716

[13] Costello N, McKenna J, Sutton L, et al. Using contemporary behavior change science to design and implement an effective nutritional intervention within professional rugby league. Int J Sport Nutr Exerc Metab. 2018;28(5):553–557. doi: 10.1123/ijsnem.2017-0298 29345174

[14] Cane J, O’Connor D, Michie S. Validation of the theoretical domains framework for use in behaviour change and implementation research. Implement Sci. 2012;7(1):1–17. doi: 10.1186/1748-5908-7-37 PMC3483008 22530986

[15] Bentley MRN, Patterson LB, Mitchell N, et al. Athletes perspectives on the enablers and barriers to nutritional adherence in high-performance sport. Psychol Sport Exerc. 2021;52:101831. doi: 10.1016/j.psychsport.2020.101831

[16] Heaney S, O’Connor H, Naughton G, et al. Towards an understanding of the barriers to good nutrition for elite athletes. Int J Sports Sci Coach. 2008;3(3):391–401. doi: 10.1260/174795408786238542

[17] Smart LR, Bisogni CA. Personal food systems of male college hockey players. Appetite. 2001;37(1):57–70. doi: 10.1006/appe.2001.0408 11562158

[18] Stokes EG, Hughes R, Shaw DM, et al. Perceptions and determinants of eating for health and performance in high-level male adolescent rugby union players. Sports. 2018;6(2):49. doi: 10.3390/sports6020049 29910353 PMC6027549

[19] Carter J, Lee DJ, Ranchordas MK, et al. Perspectives of the barriers and enablers to nutritional adherence in professional male academy football players. Sci Med Footb. 2023;7(4):394–405. doi: 10.1080/24733938.2022.2123554 36082957

[20] Birkenhead KL, Slater G. A review of factors influencing athletes’ food choices. Sports Med. 2015;45(11):1511–1522. doi: 10.1007/s40279-015-0372-1 26243016

[21] Henriksen K, Stambulova N. Creating optimal environments for talent development: a holistic ecological approach. In: Baker J; Cobley S Schorer J, editors. Routledge handbook of talent identification and development in sport. London: Routledge; 2017. p. 271–284.

[22] Ogden J, Coop N, Cousins C, et al. Distraction, the desire to eat and food intake. Towards an expanded model of mindless eating. Appetite. 2013;62:119–126. doi: 10.1016/j.appet.2012.11.023 23219989

[23] Shepherd J. Young people and healthy eating: a systematic review of research on barriers and facilitators. Health Educ Res. 2005;21(2):239–257. doi: 10.1093/her/cyh060 16251223

[24] Tsoufi A, Maraki M, Dimitrakopoulos L, et al. The effect of professional dietary counseling: elite basketball players eat healthier during competition days. J Sports Med Phys Fitness. 2017;57(10):1305–1310. doi: 10.23736/S0022-4707.16.06469-0 27232559

[25] Esparza-Ros F, Vaquero-Cristóbal R, Marfell-Jones M. International standards for anthropometric assessment (UCAM Universidad Católica de murcia, Ed.). Int Soc Adv Kinanthro. 2019. https://books.google.co.uk/books/about/International_Standars_for_Anthropometri.html?id=E4edzgEACAAJ&redir_esc=y

[26] Costello N, Deighton K, Dyson J, et al. Snap-N-Send: a valid and reliable method for assessing the energy intake of elite adolescent athletes. Eur J Sport Sci. 2017;17(8):1044–1055. doi: 10.1080/17461391.2017.1337815 28627289

[27] Fullmer S, Benson-Davies S, Earthman CP, et al. Evidence analysis library review of best practices for performing indirect calorimetry in healthy and non–critically ill individuals. J Acad Nutr Diet. 2015;115(9):1417–1446.e2. doi: 10.1016/j.jand.2015.04.003 26038298

[28] Weir JBDB. New methods for calculating metabolic rate with special reference to protein metabolism. J Physiol. 1949;109(1–2):1–9. doi: 10.1113/jphysiol.1949.sp004363 15394301 PMC1392602

[29] Ranchordas MK, Dawson JT, Russell M. Practical nutritional recovery strategies for elite soccer players when limited time separates repeated matches. J Int Soc Sports Nutri. 2017;14(1). doi: 10.1186/s12970-017-0193-8 PMC5596842 28919844

[30] Heikkilä M, Lehtovirta M, Autio O, et al. The impact of nutrition education intervention with and without a mobile phone application on nutrition knowledge among young endurance athletes. Nutrients. 2019;11(9):2249. doi: 10.3390/nu11092249 31540535 PMC6770376

[31] Garthe I, Raastad T, Refsnes PE, et al. Effect of nutritional intervention on body composition and performance in elite athletes. Eur J Sport Sci. 2013;13(3):295–303. doi: 10.1080/17461391.2011.643923 23679146

[32] Ogden J. Celebrating variability and a call to limit systematisation: the example of the behaviour change technique taxonomy and the behaviour change wheel. Health Psychol Rev. 2016;10(3):245–250. doi: 10.1080/17437199.2016.1190291 27189585

[33] Hudson JF, Cole M, Morton JP, et al. Daily changes of resting metabolic rate in elite rugby union players. Med Sci Sports Exerc. 2020;52(3):637–644. doi: 10.1249/MSS.0000000000002169 31652238

[34] Burt DG, Lamb K, Nicholas C, et al. Effects of exercise-induced muscle damage on resting metabolic rate, sub-maximal running and post-exercise oxygen consumption. Eur J Sport Sci. 2014;14(4):337–344. doi: 10.1080/17461391.2013.783628 23566074

